# Spatial structure of depression in South Africa: A longitudinal panel survey of a nationally representative sample of households

**DOI:** 10.1038/s41598-018-37791-1

**Published:** 2019-01-30

**Authors:** Diego F. Cuadros, Andrew Tomita, Alain Vandormael, Rob Slotow, Jonathan K. Burns, Frank Tanser

**Affiliations:** 1 Department of Geography and Geographic Information Science, University of Cincinnati, Cincinnati, USA; 2 Health Geography and Disease Modeling Laboratory, University of Cincinnati, Cincinnati, USA; 3 KwaZulu-Natal Research Innovation and Sequencing Platform (KRISP), Nelson R Mandela School of Medicine, College of Health Sciences, University of KwaZulu-Natal, Durban, South Africa; 4 Centre for Rural Health, School of Nursing and Public Health, University of KwaZulu-Natal, Durban, South Africa; 5 School of Nursing and Public Health, University of KwaZulu-Natal, Durban, South Africa; 6 School of Life Sciences, University of KwaZulu-Natal, Durban, South Africa; 7 Department of Genetics, Evolution & Environment, University College, London, United Kingdom; 8 Department of Psychiatry, University of KwaZulu-Natal, Durban, South Africa; 9 Institute of Health Research, University of Exeter, Exeter, United Kingdom; 10 Africa Health Research Institute, University of KwaZulu-Natal, Durban, South Africa

## Abstract

Wider recognition of the mental health burden of disease has increased its importance as a global public health concern. However, the spatial heterogeneity of mental disorders at large geographical scales is still not well understood. Herein, we investigate the spatial distribution of incident depression in South Africa. We assess depressive symptomatology from a large longitudinal panel survey of a nationally representative sample of households, the South African National Income Dynamics Study. We identified spatial clusters of incident depression using spatial scan statistical analysis. Logistic regression was fitted to establish the relationship between clustering of depression and socio-economic, behavioral and disease risk factors, such as tuberculosis. There was substantial geographical clustering of depression in South Africa, with the excessive numbers of new cases concentrated in the eastern part of the country. These clusters overlapped with those of self-reported tuberculosis in the same region, as well as with poorer, less educated people living in traditional rural communities. Herein, we demonstrate, for the first time, spatial structuring of depression at a national scale, with clear geographical ‘hotspots’ of concentration of individuals reporting new depressive symptoms. Such geographical clustering could reflect differences in exposure to various risk factors, including socio-economic and epidemiological factors, driving or reinforcing the spatial structure of depression. Identification of the geographical location of clusters of depression should inform policy decisions.

## Introduction

Mental health is a major global public health concern^1,2^, with the Global Burden of Disease Study highlighting the full extent of depressive illness as a high impact disorder that is commonly comorbid with other mental and physical diseases, and that has major public health implications^3^. Depression is the single largest contributor to global disability (7.5% of all years lived with disability), with more than 300 million cases worldwide in 2015^3^. Importantly, while 80% of the disease burden due to depression is located in low- and middle-income countries (LMICs), only 10% of these sufferers access effective treatments (reduced to 1 in 27 sufferers in low-income countries)^4,5^. In South Africa, an estimated 9.8% of the adult population experience major (clinical) depression at some point in their life^6,7^, and despite treatment being available, only 25% have sought treatment and care for a mental disorder (such as depression) within 12 months^8^. Therefore, reducing the treatment gap remains a significant challenge in the South Africa context.

South Africa is a nation in transition, undergoing rapid industrialization and urbanization, while continuing to grapple with the legacy of Apartheid — a discriminatory social system that institutionalized racial, economic and land inequality^9^. Deeply entrenched inequalities, poverty, and high rates of unemployment remain after the transition to democracy in 1994^10^. As a result, South Africa has one of the world’s highest GINI coefficients (an index intended to represent the income or wealth distribution of a nation’s residents, and is the most commonly used measure of inequality)^11^. Likewise, South Africa has a unique epidemiological landscape, in which infectious diseases, such as HIV and tuberculosis (TB), have severely affected the South African population, especially the poor^11–14^. TB remains a national crisis in South Africa, which is one of seven countries responsible for the majority of the world’s disease burden^12^, where it remains the leading cause of death^15^. TB and HIV-TB coinfection have been found to increase the risk of depression and other mental disorders^16^. TB infected individuals may experience discrimination, and may have more economic difficulties during the course of isolation or treatment, boosting the emergence of depressive symptoms^17^. Moreover, the evidence suggest a causative role of systemic infection inflammatory mediators, such as interleukin and interferon, which influence the microglial environment and neuronal activity, resulting in depression^18^.

The socio-behavioral and epidemiological features observed in South Africa generate a challenge for both understanding the epidemiological characteristics of depression, and implementing effective control interventions aimed to alleviate this growing epidemic^19^. Therefore, novel and innovative approaches are needed to understand the challenge of depression disorders in this unique epidemiological and socio-economic context.

Spatial epidemiology has emerged as a promising approach for understanding the distribution and patterns underlying the processes and drivers of an epidemic. This approach has been extensively used to study communicable and non-communicable diseases, and could provide important insights into mental health disorders, such as schizophrenia and depression^20–22^. In recent years, several studies have underscored the spatial structure of mental disorders that are partly driven by factors distributed unevenly in space^23,24^. These studies highlight the role of spatial epidemiology in understanding the contextual determinants of mental disorders. Such approaches could support the development of strategic programs that are required to promote mental health, prevent mental illnesses, reduce treatment gaps, and develop and maintain effective and safe mental health services in those areas where they are needed most. To date, however, studies have only been conducted in neighborhoods or cities, and the spatial structure of mental disorders at large geographic scales, such as national level, is unknown. The study of the spatial structure of mental disorders at this large scale is particularly important to match with and inform public health interventions or investments for improvement planned at a national or provincial level.

Against this background, using a large ongoing national representative survey conducted in South Africa, we present the first assessment of the spatial structure of depression at national level. Our primary aim was to identify the geographical locations (hotspots) where the burden of depression is concentrated. As we were also interested in identifying the drivers within any particular hotspot, our secondary aim was to conduct an analysis at the ecological level to examine the association between the spatial structure of depression and socio-economic, behavioral and burden of infectious diseases, such as TB.

## Results

### Spatio-temporal clustering analysis of existing cases of depression

**Figure 1:**
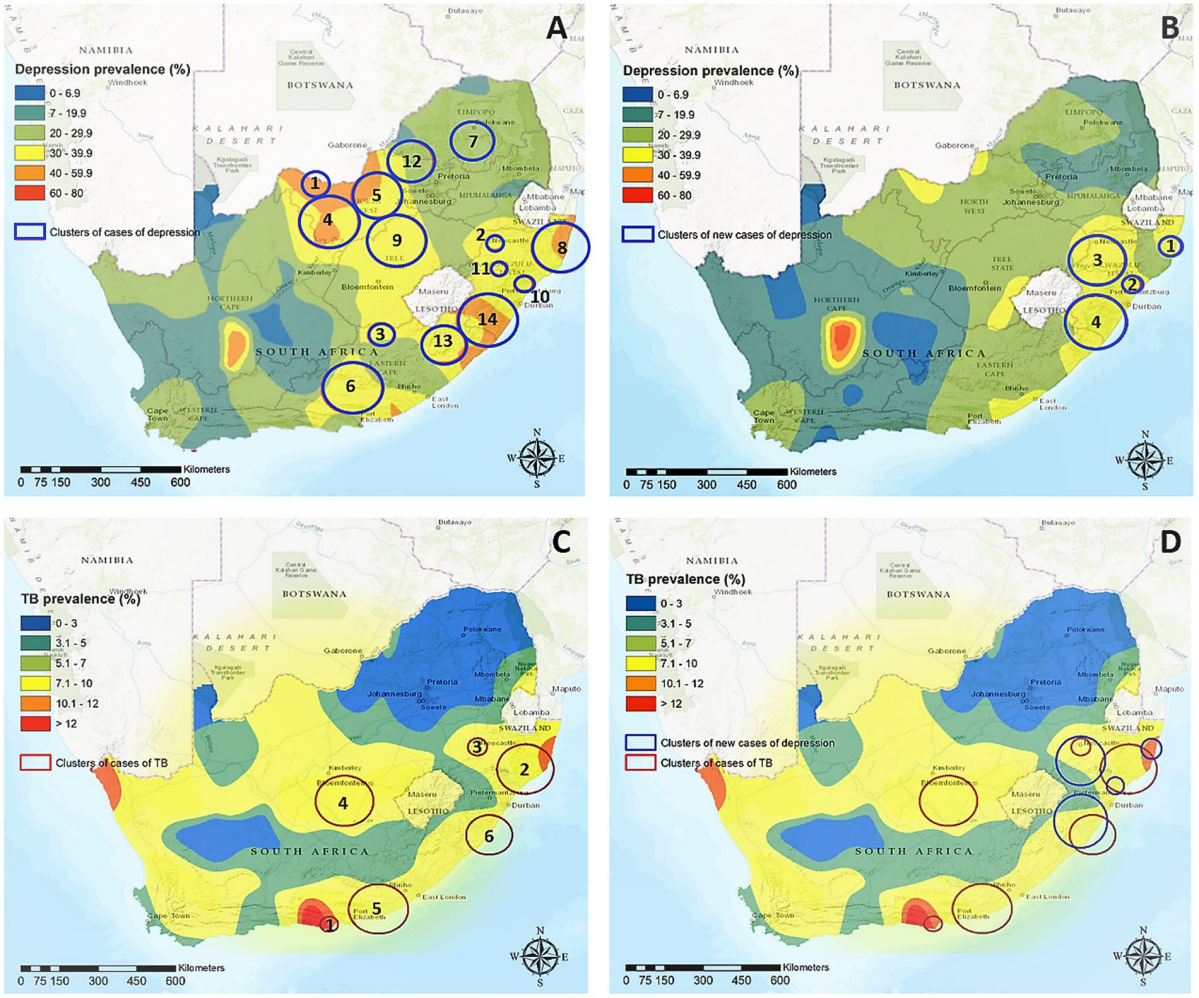
In (**A**) Continuous surface map of the percentage of existing cases of depression and the location of spatio-temporal clusters of existing cases of depression; (**B**) continuous surface map of the percentage of new cases of depression and the location of spatial clusters of new cases of depression; (**C**) Continuous surface map of the percentage of self-reported cases of tuberculosis (TB) infection and the location of spatio-temporal clusters of existing TB cases. (**D**) Geographical overlap between clusters of new cases of depression (blue circles), and clusters of self-reported TB infection (red circles). Numbers in the clusters indicates the strength of the clustering as indicated in Tables 1 and 2. Continuous surfaces of depression prevalence were generated using the Kernel interpolation algorithm using a radius of 30 km. Maps were created using ArcGIS® software by Esri version 10.3^44^ (http://www.esri.com/), and Esri World Topographic basemaps (http://www.esri.com/data/basemaps)^45^.

A total of 14,097 existing cases of depression were reported in the three survey waves conducted during 2008 to 2012. Spatio-temporal scan statistics analysis identified 14 significant clusters of depression (*P* < 0.05) in South Africa (Table 1). Most reported clusters were located in the northern and eastern part of the country, among the North West, Free State, Eastern Cape, and KwaZulu-Natal Provinces (Fig. 1A). Relative risk of depression within these clusters ranged from 1.44 in a cluster located among the Eastern Cape and KwaZulu-Natal Provinces, to 2.32 in a cluster located in the North West Province, where the prevalence of depression was 64%. The 14 clusters covered only 19.7% of the total area of South Africa, but contained 4,886 (34.7%) of reported cases of depression.

**Table 1:** Description of the spatio-temporal clusters of existing cases of depression and tuberculosis (TB) in South Africa.

	Cluster	Area (km2)	Time Frame	Observed number of cases	Expected number of cases	Strength of the clustering*	P-Value
Depression	1	6,615	2008–2010	47	20	2.3	<0.001
	2	2,357	2010–2012	571	269	2.2	<0.001
	3	5,778	2010–2012	38	18	2.1	<0.03
	4	31,086	2008–2010	235	113	2.1	<0.001
	5	22,420	2008–2010	186	91	2.0	<0.001
	6	28,517	2008–2010	59	31	1.9	<0.001
	7	16,459	2008–2010	212	112	1.9	<0.001
	8	27,274	2008–2010	253	141	1.8	<0.001
	9	29,422	2008–2010	175	98	1.8	<0.001
	10	2,826	2010–2012	241	144	1.7	<0.001
	11	2,090	2010–2012	242	153	1.6	<0.001
	12	19,152	2008–2010	231	146	1.6	<0.001
	13	15,518	2010–2012	452	310	1.5	<0.001
	14	30,526	2010–2012	1944	1408	1.4	<0.001
TB	1	2,714	2008–2012	44	10	4.4	<0.001
	2	30,279	2010–2012	34	10	3.3	<0.001
	3	3,481	2008–2012	91	39	2.4	<0.001
	4	29,361	2010–2012	64	28	2.4	<0.001
	5	29,483	2008–2012	247	151	1.7	<0.001
	6	18,762	2008–2012	143	88	1.7	<0.001

*Strength of the clustering estimated as the relative risk of depression within the cluster versus outside the cluster.

### Spatial clustering analysis of new cases of depression

The analysis of baseline characteristics of the depression incidence cohort (i.e. repeat-testers having no depressive symptoms at baseline) dataset (Table S1 in Supplementary Materials) indicated that half of study participants were female (53.3%) and younger than 35 years old (58.4%). The largest racial group was Black African (78.6%), approximately half were single (54.6%), and not employed (59.2%). The majority had completed at least a high school equivalent level of education (93.3%), and most lived in urban areas (60.7%).

From the 11,161 individuals included in the analysis that reported no symptoms of depression at baseline, 2,332 (20.89%) reported symptoms of depression in a following survey (identified as new case of depression) during the period of the study. Scan statistics analysis identified four significant spatial clusters of new cases of depression in South Africa (Table 2). Clusters of onset of depression were located at the eastern part of the country, among KwaZulu-Natal and Eastern Cape Provinces (Fig. 1B). These four clusters included 20% of the individuals in the cohort showing no symptoms at baseline, but manifested 791 (33.9%) of the new cases of depression, while enclosing only 5.2% of the total area of South Africa. In two of these clusters located in KwaZulu-Natal Province (cluster 1 and 2), new cases of depression were reported for more than 40% of the survey participants included in the cluster who showed no symptoms at baseline (Table 2).

**Table 2:** Description of the geographical clusters of new cases of depression in South Africa.

Cluster	Area (km^2^)	Observed number of cases	Expected number of cases	Strength of the clustering*	P-Value
1	4,092	23	10	2.5	0.04
2	2,940	86	38	2.3	<0.001
3	26,519	368	207	1.9	<0.001
4	30,961	328	221	1.6	<0.001

*Strength of the clustering estimated as the relative risk of depression within the cluster versus outside the cluster.

### Cluster description

In summary (Table 3), individuals reporting being TB positive significantly influenced depression clustering, these individuals having more than 50% additional likelihood of being located within the clusters of new cases of depression (odds ratio [OR] = 1.58; 95% confidence interval [CI] 1.05–2.37; *P* = 0.028). Likewise, being poorer, less educated (completed primary school only), single, Black African, and living in a traditional tribal authority setting, were also identified as factors that increased the likelihood of being located within spatial clusters of new cases of depression.

**Table 3:** Unadjusted and adjusted results: Factors associated with clusters of new cases of depression.

	Bivariate Model			Multivariate Model		
	OR	CI	P-value	OR	CI	*P*-value
*T.B. Positive Status*	1.75	1.18–2.57	0.005	1.58	1.05–2.37	0.028
*Household Income Quintile (Ref:* 3*rd)*						
1st	1.11	0.90–1.36	0.334	0.78	0.63–0.98	0.029
2nd	1.05	0.85–1.29	0.644	0.89	0.72–1.11	0.294
4th	0.51	0.39–0.65	<0.001	0.68	0.52–0.89	0.006
5th	0.33	0.25–0.47	<0.001	1.16	0.85–1.60	0.349
*Age (Ref:* 15–19*)*						
20–24	0.72	0.57–0.91	0.005	0.86	0.67–1.10	0.234
25–29	0.71	0.54–0.93	0.013	0.98	0.72–1.32	0.888
30–34	0.50	0.38–0.65	<0.001	0.86	0.62–1.18	0.339
35+	0.46	0.39–0.55	<0.001	0.77	0.58–1.02	0.064
*Education (Ref: Completed Primary School)*						
Completed High School	0.52	0.42–0.64	<0.001	0.51	0.39–0.67	<0.001
Beyond High School	0.27	0.21–0.35	<0.001	0.42	0.31–0.58	<0.001
*Female*	1.04	0.89–1.19	0.641	1.02	0.87–1.20	0.759
*Marital Status (Ref: Married)*						
Divorced/widow/separated	1.28	0.95–1.72	0.1	1.02	0.74–1.41	0.902
Single	2.31	1.95–2.75	<0.001	1.45	1.15–1.83	0.002
*Race (Ref: Black)*						
Colored	0.02	0.01–0.06	<0.001	0.06	0.02–0.19	<0.001
Asian/Indian	0.38	0.13–1.11	0.075	1.14	0.33–3.91	0.832
White	0.03	0.01–0.12	<0.001	0.17	0.04–0.67	0.011
*Employed*	0.52	0.445–0.615	<0.001	1.07	0.88–1.29	0.481
*Civic Engagement*	0.59	0.49–0.69	<0.001	0.59	0.49–0.70	<0.001
*Development (Ref: Tribal authority)*						
Rural formal	0.22	0.17–0.28	<0.001	0.26	0.18–0.36	<0.001
Urban formal	0.07	0.05–0.08	<0.001	0.10	0.08–0.13	<0.001
Urban informal	0.50	0.38–0.67	<0.001	0.54	0.41–0.73	<0.001

### Spatio-temporal clustering analysis of existing cases of self-reported TB infection

A total of 2,287 existing cases of TB were reported in the three different survey waves conducted during 2008 to 2012. Spatio-temporal Scan statistics analysis identified six significant spatio-temporal clusters of TB in South Africa (Table 1). Similar to the clusters of onset of depression, most of the TB clusters were located at the eastern part of the country among the Free State, Eastern Cape, and KwaZulu-Natal Provinces (Fig. 1C). Relative risk of depression within these clusters ranged from 1.66, to 4.38, both areas being located in KwaZulu-Natal Province. A total of 332 cases of self-reported TB infection (14.5% of the cases) were reported within the six clusters identified, which cover 9.3% of the total area of South Africa.

## Discussion

The burden of depression is clearly clustered in specific geographic locations in South Africa. Using state of the art methodology for clustering detection, and data from a nationally representative survey conducted in different years in South Africa, we identified several geographical ‘hotspots’ of existing (prevalent) cases of depression in the northern and eastern parts of South Africa. Importantly, we also identified clear geographical clusters of new (incident) cases of depression situated mainly in KwaZulu-Natal Province, within which more than 40% of the participants reported symptoms of depression during the time period covered by the surveys (2008–12), amongst the highest rates ever identified in any community context^5^.

Moreover, to our knowledge, this is the first report of the geographic distribution of mental health being associated with an infectious disease (TB) epidemic. Individuals who reported being infected with TB had significantly higher odds of belonging to a hotspot of new cases of depression. This was after controlling for other important socio-economical and demographical risk factors of depression, identified in previous work in sub-Saharan African settings, as well as in studies assessing the geographical risk factors of depression at smaller scales^20,25^. Likewise, the clusters of new cases of depression overlapped with those of TB self-reporting (Fig. 1D), particularly in the KwaZulu-Natal Province. South Africa is characterized by a particular epidemiological landscape, enduring one of the largest HIV epidemics in the world, which has facilitated the emergence of other epidemics, such as TB^14^. ‘Hotspots’ of depression identified geographically overlap with areas where the highest burden of HIV is concentrated, particularly in KwaZulu-Natal Province, which has one of the highest HIV and TB prevalence rates, and where the two infections collide^26^.

Several studies have assessed the impact of life-threatening diseases, such as TB and HIV, on the mental health of infected individuals^27–29^. Depression is one of the most common causes for psychological evaluation and treatment of people living with HIV infection^27^. The stress and physical deterioration caused by diseases such HIV and TB have large impacts on the mental health of an individual^30^. Factors such as feelings about social discrimination and stigma, stress, constant need for health care, potential lack of family support, and response to medications, are among those that could drive HIV infected individuals to development depressive symptoms^31^.

However, mounting evidence suggests that depression may be predictive of human morbidity and mortality in a wide range of diseases^32^. Depression may alter cellular immunity, and may therefore contribute to disease progression in certain immune diseases, such as HIV infection^33^. For example, depression may have a negative impact on innate immunity by decreasing the natural killer T-cell activity, leading to an increase in activated CD8 T lymphocytes and viral load^28^. Moreover, depression can be associated with lower adherence for treatment of chronic conditions, including therapeutic regimen to TB or HIV^17^, generating a synergistic relationship between depression and infectious disease epidemics, such as HIV, and emerging as a major public health challenge that needs to be addressed.

The geographical approach implemented in our study contributes to a deeper insight into the contextual determinants of mental disorders, such as depression, in a sub-Saharan African setting. Clusters of onset of new depression are indicated by poorer, less educated people living in traditional rural communities, adding to their burden of inequality and the challenge of social development. Furthermore, the burden of depression is located in areas where epidemics of infectious diseases such as TB and HIV are concentrated in South Africa, which highlights a major compounding challenge for the health care system of the country. The large TB and HIV epidemics that are currently affecting South Africa might have a direct or indirect impact on the mental health of its population, paving the way for the establishment of a major depression epidemic. Likewise, depression might affect HIV or TB treatment outcomes associated with poor adherence for treatment of these conditions. Understanding the potential association between infectious agents, such as HIV or TB, and the development of depression could provide means to improve both depression outcomes and prevention in South Africa. This strategy becomes particularly relevant in provinces such as KwaZulu-Natal, one of the area’s most severely affected by the HIV and TB epidemics, with large poor, traditional, rural communities, and where an emerging epidemic of depression, indicated by the clustering of new cases of depression, is concentrated.

Despite the strengths of our study, a number of limitations are worth noting. First, despite the fact that a well-developed method for scoring symptoms was used, this study relies on self-reported symptoms of depression and not on a formal diagnosis of a depressive disorder. Analysis of factors associated with the geographical clustering of depression was constrained by the availability of these factors in the surveys. For instance, factors such as migration, environmental characteristics, such as pollution or toxic factors, and stress, among other health-related variables, were not collected by the surveys, and were therefore not available for the analysis. Likewise, TB data were collected as self-reported status of the infection, and, with a self-reported measure such as TB, we anticipate some ascertainment error in TB status. However, such error would bias the result towards the null-hypothesis of no link to TB, and could not explain a spurious positive association between TB and the clustering of depression. Moreover, HIV-related information, such HIV status, or behavioral data associated with the risk of HIV infection, were not collected. Importantly, is not possible to infer associations at an individual level from the geographical overlap observed between depression and infectious diseases, such HIV and TB.

Additionally, the analyses conducted here did not identify a direct causal relationship between the spatial structure of depression and infectious diseases, such as TB. The link observed between depression and TB could have been modulated by external drivers exacerbating each disease outcome independently. Some factors that could have an impact on both conditions, and feed into causal pathways, including food security, malnutrition, over-crowding, access to healthcare and HIV, among others, were not included. Therefore, the interpretation of the potential direct link between the spatial clustering of depression and infectious diseases, such as TB, should be interpreted with caution. An in-depth exploration of the potential association between infectious diseases and depression, as well as other factors that could be driving the depression epidemic in South Africa, is warranted. Despite these limitations, this is the first study, to our knowledge, that investigates the spatial structure of depression at a national scale. Using a nationally representative dataset, we found that individuals reporting depressive symptoms are concentrated in geographical ‘hotspots’ in South Africa. Furthermore, we were able to identify hotspots of onset of depression, reflecting a changing landscape of future burden of disease.

**Figure 2:**
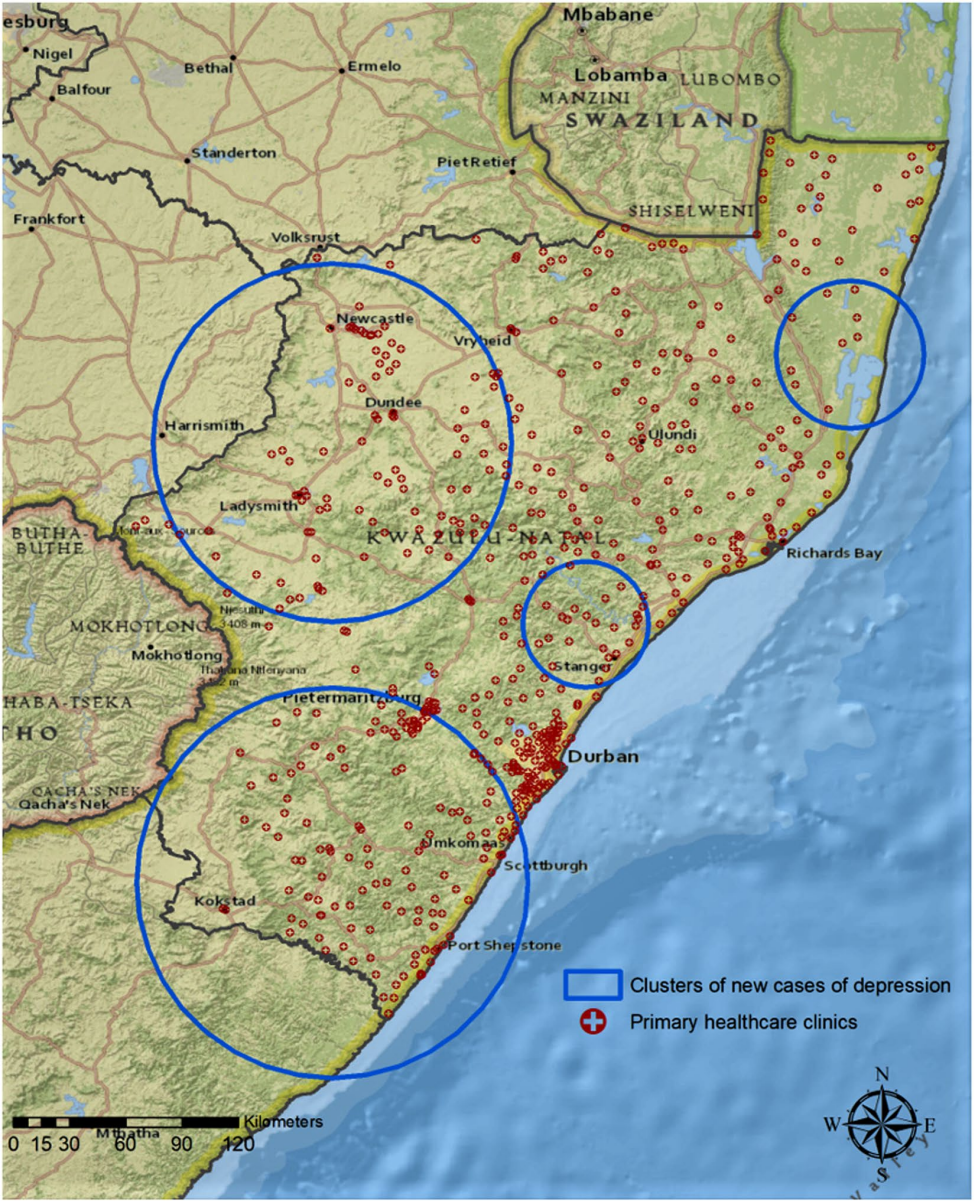
Location of primary healthcare clinics in KwaZulu-Natal, and the location of spatial clusters of new cases of depression. Primary healthcare clinic locations were obtained from the Kwazulu-Natal Department of Health^46^. Note that, for the two clusters that extend outside of Kwazulu-Natal, only clinics within Kwazulu-Natal are depicted. Maps were created using ArcGIS® software by Esri version 10.3 (http://www.esri.com/), and Esri World Topographic basemaps (http://www.esri.com/data/basemaps)^45^.

The identification and geographical location of these ‘hotspots’ of depression should play a key role for enabling well-informed policy decisions. It is clear from our results that provinces in South Africa face different epidemic intensities and onset of depression, and therefore need to plan and implement measures that are tailored to their circumstances to be most effective at reducing their burden of disease, especially complex interaction with social development needs. For example, we have already demonstrated elsewhere that a distance of >15 km from a primary healthcare facility significantly increases the onset of depression, these high distances being associated with such at risk communities^34^. We calculated that the density of primary healthcare clinics within the clusters of new cases of depression in the KwaZulu-Natal Province is considerably lower than the density of primary healthcare clinics outside these areas (0.47 clinics/100 km^2^ within the clusters compared to 0.82 clinics/100 km^2^ outside these areas; Fig. 2). Reduced access to primary healthcare clinics within the clusters of depression might play an important role in driving the burden of depression. This highlights the need for expanding primary healthcare services to appropriately integrate mental healthcare interventions in these geographical areas where the disease burden is increasing most rapidly. Failure to reduce inequalities in service provisioning, such as distance to nearest clinic, greatly exacerbates inequalities in human wellbeing outcomes in such disadvantaged communities.

In summary, South Africa is not characterized by a single epidemic of depression, with marked heterogeneities in its distribution at fine scales within the country, potentially driven by several socio-economic and epidemiological cofactors. Our findings highlight how, rather than having a national epidemic of depression in South Africa, there are spatially localized micro-epidemics. We characterized these micro-epidemics quantitatively, thus paving the way for a targeted approach towards prevention programming by focusing on areas of greatest relevance and need. This provides for optimizing resources and maximizing the impact of interventions, in terms of both care services to treat existing cases of depression, and interventions to prevent new cases of depression. This evidence may be critical in allocating resources and organizing healthcare services aimed at addressing the depression epidemic in South Africa, for example, addressing the inequality in access to primary healthcare facilities. Likewise, this information can assist with the development of effective tools required to promote mental health and maintain efficient and safe mental health services. These could be allocated in geographical areas where the burden of this mental disorder is concentrated, and where there may be strong interactions with other elements of public health burden, such as infectious or non-communicable diseases, especially those associated with food insecurity and diet. The high heterogeneity that we identified in the distribution of the health burden across the highly unequal socio-economic landscape of South Africa is likely to prevail in other developing countries, especially where health systems are challenged with expanding multiple disease burdens, compounded by environment challenges, such as poverty and food insecurity. Understanding geographical patterns below the country level is critical to informing effective and efficient health system interventions where resources are highly limited.

## Methods

### Data sources

We obtained the data from the South African National Income Dynamics Study (SA-NIDS), a longitudinal panel survey of a nationally representative sample of households^35^. Our study included SA-NIDS data from three waves; wave 1, conducted in 2008, wave 2 in 2010, and wave 3 in 2012. The survey method of the SA-NIDS is described in its published report^36^. As also described in our previous report^37^, the SA-NIDS employs a stratified, two-stage cluster sample design to obtain a nationally representative sample of South African households. In the first stage, 300 of 400 Primary Sampling Units (PSU) from Statistics South Africa’s Master Sample were randomly selected for inclusion. In stage two, two clusters of 12 dwelling units each were selected from each PSU (24 dwelling units per PSU). All consenting household members (adults and children) at the selected dwelling units were included in SA-NIDS.

Questionnaires were available and administered in South Africa’s 11 official languages, the main measure of the study being depression symptomatology, which was obtained from the adult questionnaires from the three different waves. To obtain this data, we used the 10-item abridged version of the Center for Epidemiologic Studies Depression Scale (CES-D) to assess depression symptomatology^38^.

Using data from the three waves, we constructed two datasets: 1) consisted of 25,961 unique participants across the three waves who responded to the questionnaire for depression assessment, and 2) entailed constructing an incidence cohort dataset using the three survey waves. The inclusion criterion for this second dataset was adult repeat-testers, defined as study participants with a minimum of two depression risk assessments between waves 1–3, but with no depressive symptoms at baseline (which could have been either wave 1 or 2, depending on when that individual was first sampled). With the purpose of constructing an incident cohort, while reducing the possibility of reverse causation, study participants with a positive screen for significant depressive symptoms upon first entry to SA-NIDS were excluded. The incident cohort consisted of 11,161 repeat-testers. After data filtering, we conducted descriptive statistics to summarize the baseline socio-demographic, epidemiological and household factors of the incidence cohort dataset generated. Prevalence of significant depressive symptoms increased to 15.9% (1,656 individuals) in wave 2 and 20.8% (1,896 individuals) in wave 3. Further description of the data sources is included the Supplementary Material.

Ethical approval for the NIDS parent study was granted by the University of Cape Town, Commerce Faculty Ethics Committee, and consent was obtained from each study participant^36^. Our study obtained permission from the University of KwaZulu-Natal Biomedical Research Ethics Committee (BE 111/14) to conduct this current secondary data investigation.

### Spatio-temporal clustering analysis of existing cases of depression

For this analysis, the first dataset constructed, which included all unique individuals across the three waves, was analyzed to identify geographical clusters of depression cases that persisted during at least two different consecutive waves. Using this dataset, spatio-temporal clusters of cases of depression were identified using a spatial scan statistical analysis^39^, identified in the SaTScan software^40,41^. A further description of the spatio-temporal clustering analysis is included the Supplementary Material.

### Spatial clustering analysis of new cases of depression

Using the second incidence cohort dataset that was constructed, a purely spatial clustering analysis (as the temporal domain was already included in the cohort onset assessment) using SaTScan was conducted to identify geographical clusters of new cases of depression. Clusters with a *P* < 0.05 were identified as statistically significant and were examined for additional socio-economical and epidemiological description. A description of the temporal clustering analysis is included in Supplementary Material.

### Spatio-temporal clustering analysis of existing cases of self-reported TB infection

To evaluate our hypothesis regarding the potential spatial association between the burden of depression and a major infectious disease, we explored the spatial structure of self-reported TB infection in South Africa. Measures of TB burden were included in NIDS as self-reported cases of infection to the question: “have you ever been told by a doctor, nurse or health care professional that you have TB”. Similar to the methodology used for detecting the spatio-temporal clustering of existing cases of depression described above, the first dataset constructed, which included all unique individuals across the three waves, was analyzed to identify the spatio-temporal clustering of self-reported cases of TB using the same spatial scan statistical analysis to identify the geographical clusters of cases of TB that persisted during at least two different consecutive waves.

Continuous surface maps of the percentage of existing and new cases of depression and TB were generated using a standard kernel interpolation technique^42^. These measures were computed by means of a moving two-dimensional Gaussian kernel of a 30-km search radius. First, all participants were located to the exact household of residence, and the measurements (existing cases and new cases of depression, or TB) superimposed on a geographic representation of the study area consisting of a grid of 1 km × 1 km pixels. Next, the kernel moved systematically across the grid and calculated a Gaussian-weighted estimate of the different measures for the unique neighborhood around each and every pixel on the grid. Final maps were generated by over-imposing the location of the clusters identified for the different measures (existing cases of depression, new cases of depression, and TB) over their corresponding continuous surface maps.

### Cluster description

We investigated the potential socio-economic, demographic and epidemiologic factors associated with the spatial clustering of new cases of depression that were detected from the purely spatial clustering analysis of the second dataset. Known factors that have been previously associated with depression included in the analysis were: self-reported TB status, gender, marital status, race/ethnicity, age, education attainment, employment status, household civil participation, household income and geographic typology of residence. Data on gender, marital status, education, age, race or ethnicity, and employment status were obtained from the adult questionnaires, and geographic typology of residence, household civil participation and household income data were obtained from the household questionnaires.

The association between these factors and the likelihood of belonging to a cluster of new cases of depression was assessed using a logistic regression model, in which the main outcome was being located within such a cluster. As the SA-NIDS was designed as a complex survey, we adjusted the analysis using post-stratification weights for the three waves, which allowed our results to be better representative of the South African population. These post-stratification weights for the three waves were based on the age-sex-race distribution for the mid-year population estimates in 2008, 2010 and 2012 using estimates obtained from Statistics South Africa, their construction being described in published report^43^.

### Ethical approval and informed consent

Our study obtained permission from the University of KwaZulu-Natal Biomedical Research Ethics Committee (BE 111/14) to conduct the study. Ethical approval for NIDS was granted by the University of Cape Town (UCT) Commerce Faculty Ethics Committee^36^. The NIDS data collectors administered a written informed consent process with all participants, and only proceeded with interviews once this process was complete and they were satisfied that the participant fully understood all aspects of the research. All methods were performed in accordance with the relevant guidelines and regulations.

## Supplementary information

Supplemetary materials

## Data Availability

The source of data used in this research was from the South African National Income Dynamics Study (NIDS). NIDS data can be accessed via the NIDS website (http://www.nids.uct.ac.za/). However, for this particular study, certain secure data from NIDS were used (geographical coordinates of each household). These secure data can be accessed by applying to Datafirst (www.datafirst.uct.ac.za) at the University of Cape Town.
