# Supplementary material for: Spatial structure of depression in South Africa: A longitudinal panel survey of a nationally representative sample of households
Source: Sci Rep. 2019 Jan 30;9:979. doi: 10.1038/s41598-018-37791-1 (PMC6354020; doi:10.1038/s41598-018-37791-1)
Supplement: Supplementary file 1 — Supplemetary materials [file 41598_2018_37791_MOESM1_ESM.pdf]

Supplementary Materials

Spatial structure of depression in South Africa: A longitudinal panel survey of a nationally representative sample of households

Diego F. Cuadros[1,2*], Andrew Tomita[3,4], Alain Vandormael[3,5], Rob Slotow[6,7], Jonathan K. Burns[8,9], Frank Tanser[10,5]

[1]*Department of Geography and Geographic Information Science, University of Cincinnati, Cincinnati, U.S.A.*

[2]*Health Geography and Disease Modeling Laboratory, University of Cincinnati, Cincinnati, USA*

[3]*KwaZulu-Natal Research Innovation and Sequencing (KRISP), Nelson R Mandela School of Medicine, College of Health Sciences, University of KwaZulu-Natal, Durban, South Africa*

[4]*Centre for Rural Health, School of Nursing and Public Health, University of KwaZulu-Natal, Durban, South Africa.*

[5]*School of Nursing and Public Health, University of KwaZulu-Natal, Durban, South Africa.*

[6]*School of Life Sciences, University of KwaZulu-Natal, Durban, South Africa.*

[7]*Department of Genetics, Evolution & Environment, University College, London, United Kingdom.*

[8]*Department of Psychiatry, University of KwaZulu-Natal, Durban, South Africa.*

[9]*Institute for Health Research, University of Exeter, Exeter, United Kingdom.*

[10]*Africa Health Research Institute, University of KwaZulu-Natal, Durban, South Africa.*

*To whom correspondence should be addressed. E-mail: diego.cuadros@uc.edu

Supplementary Methods

Data sources

We obtained the data from the South African National Income Dynamics Study (SA-NIDS), a longitudinal panel survey of a nationally representative sample of households[1]. Our study included SA-NIDS data from three different waves; Wave 1, conducted in 2008, Wave 2 in 2010, and Wave 3 conducted in 2012.

Households were sampled based on a stratified two-stage cluster design. For the first stage, 3,000 primary sampling units (PSUs) were selected for inclusion. During the second stage, two clusters, of 12 dwelling units each, were drawn from within each PSU (24 dwelling units per PSU). The household questionnaire, upon consent, was administered by trained fieldworkers to the oldest woman in each household, or to another household member knowledgeable about living arrangements. Every consenting household member aged 15 or older in all the sampling units participated in the adult questionnaire. The global positioning system (GPS) was used to record the geographical coordinates of each household included in the study. Household GPS coordinates from the SA-NIDS were accessed from DataFirst's Secure Data Centre at University of Cape Town. Further details related to the SA-NIDS methodology can be found elsewhere[2-4].

Questionnaires were available and administered in South Africa's 11 official languages. The main measure of the study was depression symptomatology, which was obtained from the adult questionnaires from the three different waves. Here, we used the 10-item abridged version of the Center for Epidemiologic Studies Depression Scale (CES-D) to assess depression symptomatology. Briefly, the original 20-item CES-D is a psychometrically tested instrument to screen for depression risk[5-8] that has been previously used in other South African studies[9,10]. The

CES-D is based on self-report measures that capture depression-associated symptoms during the past week. It contains four possible responses: 0 = rarely or none of the time (< 1 day in the previous week); 1 = some or little of the time (1 - 2 days); 2 = occasionally or a moderate amount of time (3 - 4 days); 3 = almost or all of the time (5 - 7 days). Depression symptomatology was based on a composite score of 10 items (Cronbach's alpha = 0.73, range from 0 - 30). The higher score indicates higher risk for the disorder. For our study, a score of ten or greater was used as a cut-off to indicate significant depressive symptomatology, as previously used[6].

Spatio-temporal clustering analysis of existing cases of depression

For this analysis, the first dataset constructed that included all unique individuals across the three waves was analyzed to identify geographical clusters of cases of depression that persisted during at least two different consecutive waves. Using this dataset, spatio-temporal clusters of cases of depression were identified using a spatial scan statistical analysis[11], implemented in the SaTScan software[12,13]. Scan statistics are widely used for cluster-detection in epidemiology[14-17], social sciences[18], crime mapping[19], and, very recently, in mental health[20], among other applications. Briefly, the spatial scan statistic is a cluster-detection test able to both identify and test the statistical significance of specific geographical clusters. An advantage provided by the spatial scan statistic test is the ability to account for the uneven distributions of the sampled population[11].

For the spatio-temporal clustering analysis, the space-time permutation model examined clusters of cases of depression both spatially and temporally, by testing whether cases were significantly close in space and time[12]. Scan statistical analysis uses a scanning window that forms a cylinder

with the base representing space, while the height reflects the time period of potential clusters. Cylinders of varying radii spanned the study region and time period to identify areas where cases of depression were clustered. The cylinder varied continuously in both location and radius, thus creating and testing a very large number of distinct potential clusters. Each potential cluster was then tested using a likelihood ratio test to determine the statistical significance against the null hypothesis of spatial randomness. Clusters with a $P < 0.05$, calculated through Monte Carlo simulations (using the default value of 999 iterations), were identified as statistically significant clusters of cases of depression. After a cluster was identified, the strength of the clustering was estimated using the relative risk (RR) of cases of depression within the cluster versus outside the cluster[12].

Spatial clustering analysis of new cases of depression

Using the second dataset constructed, the incidence cohort dataset previously described, a purely spatial clustering analysis (as the temporal domain was already included in the cohort onset assessment) using SaTScan was conducted to identify geographical clusters of new cases of depression. For this analysis, Scan statistics scanned a circular window that spaned the study region. The radius of the circle was changed continuously so that it could take any value from 0 up to the default value of 50% of the sampled population inside the window. For each potential cluster, a likelihood ratio test was computed assuming that the number of new cases of depression in each circular window was an independent Bernoulli random variable. The numbers of observed and expected new cases of depression within and outside the circular window were then compared with the likelihood L_0 under the null hypothesis of spatial randomness. The circular windows with the highest likelihood ratio values were identified as potential clusters. An associated *P*-value of the statistics was then determined through Monte Carlo simulations, and

used to evaluate whether new cases of depression were randomly distributed in space or not[11]. Clusters with a $P < 0.05$ were identified as statistically significant clusters, and they were examined further for additional socio-economical and epidemiological description.

Supplementary Table

Supplementary Table S1. General characteristics of the incidence cohort dataset (N=11,161)

Variable		Percentage (%)	n
Gender	Male	46.71%	4,781
	Female	53.29%	6,380
Marital status	Married/living with partner	38.04%	3,772
	Divorced/separated/widow	7.33%	863
	Never married	54.63%	6,484
Race/ethnicity	African	78.64%	8,787
	Coloured	9.39%	1,720
	Asian/Indian	2.61%	151
	White	9.36%	503
Age category	15-19	22.92%	2,886
	20-24	12.68%	1,481
	25-29	11.73%	1,105
	30-34	11.23%	950
	35+	41.45%	4,739
Tuberculosis status	Positive	2.62%	337
	Negative	97.38%	10,801
Education	Less than high school	6.61%	1,111
	Completed high school	62.23%	7,467
	Beyond high school	31.16%	2,582
Employment status	Not employed	59.24%	7,112
	Employed	40.76%	3,953
Household civic participation	No	62.86%	7,228
	Yes	37.14%	3,908
Household income	Lowest 20%	22.02%	2,671
	Low/Middle 20%	16.93%	2,310
	Middle 20%	17.31%	2,164
	Middle/High 20%	17.49%	2,014
	Highest 20%	26.25%	2,002
Development (typology)	Rural	7.39%	1,158
	Tribal authority	31.91%	4,681
	Urban formal	50.13%	4,620
	Urban informal	10.57%	702

References

1. Southern Africa Labour and Development Research Unit. National Income Dynamics Study. Cape Town: Southern Africa Labour and Development Research Unit. Cape Town: DataFirst, 2016.

2. Southern Africa Labour and Development Research Unit. National Income Dynamics Study 2008, Wave 1. Version 6.1. Cape Town: Southern Africa Labour and Development Research Unit. Cape Town: DataFirst, 2016.

3. Southern Africa Labour and Development Research Unit. National Income Dynamics Study 2010-2011, Wave 2. Version 3.1. Cape Town: Southern Africa Labour and Development Research Unit. Cape Town: DataFirst, 2016.

4. Southern Africa Labour and Development Research Unit. National Income Dynamics Study 2012, Wave 3. Version 2.1. Cape Town: Southern Africa Labour and Development Research Unit. Cape Town: DataFirst, 2016.

5. Radloff LS. The CES-D scale: A self-report depression scale for research in the general population. *Applied psychological measurement* 1977; **1**(3): 385-401.

6. Andresen EM, Malmgren JA, Carter WB, Patrick DL. Screening for Depression in Well Older Adults: Evaluation of. *Prev Med* 1994; **10**: 77-84.

7. Björgvinsson T, Kertz SJ, Bigda-Peyton JS, McCoy KL, Aderka IM. Psychometric properties of the CES-D-10 in a psychiatric sample. *Assessment* 2013; **20**(4): 429-36.

8. Cole JC, Rabin AS, Smith TL, Kaufman AS. Development and validation of a Rasch-derived CES-D short form. *Psychological assessment* 2004; **16**(4): 360.

9. Hamad R, Fernald L, Karlan D, Zinman J. Social and economic correlates of depressive symptoms and perceived stress in South African adults. *Journal of Epidemiology and Community Health* 2008; **62**(6): 538-44.

10. Myer L, Smit J, Roux LL, Parker S, Stein DJ, Seedat S. Common mental disorders among HIV-infected individuals in South Africa: prevalence, predictors, and validation of brief psychiatric rating scales. *AIDS patient care and STDs* 2008; **22**(2): 147-58.

11. Kulldorff M. A spatial scan statistic. *Communications in Statistics - Theory and Methods* 1997; **26**(6): 1481-96.

12. Kulldorff M. SaTScan v9.0: software for the spatial and space-time statistics. National Cancer Institute. 2010.

13. Kulldorff M, Heffernan R, Hartman J, Assunção R, Mostashari F. A Space–Time Permutation Scan Statistic for Disease Outbreak Detection. *PLoS Med* 2005; **2**(3): e59.

14. Wand H, Ramjee G. Targeting the hotspots: investigating spatial and demographic variations in HIV infection in small communities in South Africa. *Journal of the International AIDS Society* 2010; **13**(1): 41.

15. Ryan J, Mbui J, Rashid J, et al. Spatial clustering and epidemiological aspects of visceral Leishmaniasis in two endemic villages, Baringo District, Kenya. *American Journal of Tropical Medicine and Hygiene* 2006; **74**(2): 308 - 17.

16. Kulldorf M, Song C, Gregoria D, Samociuk H, DeChello L. Cancer map patterns: are they random or not? *Am J Prev Med* 2006; **30**: s37 - s49.

17. Cuadros D, Awad S, Abu-Raddad L. Mapping HIV clustering: a strategy for identifying populations at high risk of HIV infection in sub-Saharan Africa. *International Journal of Health Geographics* 2013; **12**(1): 28.

18. Jones P, Gunnell D, Platt S, et al. Identifying probable suicide clusters in Wales using national mortality data. *PLoS One* 2013; **8**(8): e71713.

19. Malleson N, Andresen MA. Spatio-temporal crime hotspots and the ambient population. *Crime science* 2015; **4**(1): 1-8.

20. Ruiz-Grosso P, Miranda JJ, Gilman RH, et al. Spatial distribution of individuals with symptoms of depression in a periurban area in Lima: an example from Peru. *Annals of epidemiology* 2016; **26**(2): 93-9. e2.
